# Vitamin D Deficiency and Clinical Outcomes in Adult Burn Patients: A Systematic Review and Meta-Analysis

**DOI:** 10.7759/cureus.99123

**Published:** 2025-12-13

**Authors:** Daniel Madarshahian, Anjana Kaur, Jvalant Parekh, Yvonne Wilson

**Affiliations:** 1 Plastic Surgery, Birmingham Women's and Children's NHS Foundation Trust, Birmingham, GBR; 2 Plastic Surgery, Birmingham Women’s and Children’s NHS Foundation Trust, Birmingham, GBR; 3 Plastic and Reconstructive Surgery, Queen Elizabeth Hospital Birmingham, Birmingham, GBR

**Keywords:** bacteremia, burn injuries, clinical outcomes, critical care, vitamin d deficiency

## Abstract

Vitamin D deficiency is highly prevalent among burn patients and may impair immune function, inflammation control, and wound healing. To clarify its clinical impact, we conducted a systematic review and meta-analysis of observational studies evaluating outcomes stratified by baseline vitamin D status in adult burn patients. Six endpoints were analyzed: wound infection or cellulitis, bacteremia or septicemia, intensive care unit (ICU) admission, mechanical ventilation, ICU length of stay (LOS), and total hospital LOS. Random-effects models generated pooled odds ratios (ORs) and weighted mean differences (WMDs) with 95% CI. Five studies comprising 1,814 patients met the inclusion criteria. Vitamin D sufficiency was associated with significantly reduced odds of bacteremia or septicemia (OR 0.21; 95% CI 0.10-0.43; p<0.0001) and shorter ICU (WMD -5.5 days; p=0.04) and hospital LOS (WMD -5.7 days; p=0.003). A borderline reduction in mechanical ventilation was also observed (OR 0.55; p=0.05), whereas ICU admission and wound infection outcomes showed non-significant trends favoring sufficiency. Heterogeneity varied across outcomes, with the bacteremia analysis demonstrating no heterogeneity (I²=0%). Vitamin D deficiency appears associated with increased systemic infection risk and prolonged hospitalization following burn injury, supporting further prospective trials to evaluate vitamin D supplementation in burn care.

## Introduction and background

Vitamin D deficiency is a significant public health issue in the UK and across Europe, affecting both adults and children. It has been associated with a wide range of adverse health outcomes, including rickets, impaired growth in children, osteomalacia, cardiovascular dysfunction, and compromised immune regulation in adults [1-4]. A UK government review in 2022 estimated that one in six adults and nearly 20% of children are deficient in vitamin D, with disproportionately higher rates in Black, Asian, and Minority Ethnic populations due to reduced cutaneous synthesis and lower dietary intake [5].

Vitamin D plays a well-documented role in calcium homeostasis and bone metabolism, but its broader physiological effects are increasingly recognized. It has immunomodulatory, anti-inflammatory, and epithelial repair functions, mediated through vitamin D receptors on keratinocytes, fibroblasts, and immune cells. These mechanisms suggest a potential role in regulating wound healing, infection risk, and systemic inflammation [6-8]. Despite this, vitamin D screening and correction are not routinely included in standard clinical care pathways for many high-risk conditions.

Burn injuries represent one such context. Globally, burns account for approximately 180,000 deaths per year, with the majority occurring in low- and middle-income countries. Survivors of major burns frequently require prolonged hospitalization, intensive care admission, and repeated surgical intervention [9]. Nutritional depletion and a hypermetabolic state are characteristic of the post-burn period, intensifying the body’s demand for micronutrients, including vitamin D [10]. In this population, vitamin D deficiency is common, often present prior to injury and further exacerbated by post-burn dermal loss, impaired hepatic and renal activation, reduced sun exposure, and persistent inflammation [11,12]. One observational study found that nearly 80% of burn patients were vitamin D deficient [13].

Emerging evidence suggests that vitamin D status may influence clinical outcomes in burn patients. Associations have been reported between low vitamin D levels and increased risks of wound infection, sepsis, mechanical ventilation, and prolonged hospital and intensive care unit (ICU) stays. However, findings remain inconsistent, and there is no current consensus regarding the prognostic value of vitamin D deficiency in this context [12-16].

Given these uncertainties, a systematic review and meta-analysis are warranted to clarify the relationship between vitamin D status at the time of hospitalization and key clinical outcomes in adult burn patients. To our knowledge, this is the first meta-analysis to examine this question. The primary outcomes of interest include wound infection or cellulitis, bacteremia or septicemia, ICU admission, requirement for mechanical ventilation, ICU length of stay (LOS), and hospital LOS.

## Review

Methods

Design and Study Selection

The selection of studies, data collection, outcome synthesis, and statistical analysis were conducted in accordance with prespecified criteria outlined in a review protocol. This protocol was registered with the International Prospective Register of Systematic Reviews (PROSPERO) under the registration number CRD420251047382. The review conformed to the Preferred Reporting Items for Systematic Reviews and Meta-Analyses (PRISMA) 2020 standards [17].

For this meta-analysis, we included observational studies employing a retrospective or prospective cohort design that enrolled adult patients aged 18 years or older with burn injuries and reported serum 25-hydroxyvitamin D [25(OH)D] levels either at the time of hospital admission or shortly thereafter, before the initiation of any vitamin D supplementation. Eligible studies were required to report clinical outcomes stratified by vitamin D status, allowing for a comparative analysis between patients with sufficient versus insufficient or deficient vitamin D levels. No restrictions were applied regarding the etiology or mechanism of burn injury; included studies encompassed flame, scald, contact, electrical, and chemical burns. Similarly, no limitations were placed on total body surface area (TBSA) or burn depth. Given the limited number of available studies, an inclusive approach to burn severity and extent was adopted to enable meaningful synthesis of the data and to explore general trends in how vitamin D deficiency may influence burn-related outcomes. While physiological responses differ by burn depth and TBSA, this broader inclusion reflects the spectrum of injuries encountered in clinical settings and provides preliminary insight into vitamin D’s relevance across diverse patient presentations. Studies that met these criteria and reported at least one relevant clinical outcome were included in the final meta-analysis. Studies were excluded if they did not stratify outcomes based on vitamin D status, focused only on vitamin D metabolism or longitudinal vitamin D trends without linking them to clinical outcomes, investigated endpoints unrelated to the primary aim (such as genetic polymorphisms or chronic pain), did not include a comparator group, or involved pediatric populations or animal models. Case reports, review articles, and conference abstracts without full datasets were also excluded. These inclusion and exclusion criteria were applied to ensure that the studies selected for analysis were methodologically appropriate and addressed the clinical research question with sufficient rigor and relevance. To ensure analytical robustness, we included only those outcomes reported in at least three of the included studies. This threshold was established to allow meaningful statistical pooling and adequate assessment of between-study heterogeneity. Based on these criteria, six clinical outcomes were included in the meta-analysis: the incidence of wound infection or cellulitis, the incidence of bacteremia or septicemia, the rate of ICU admission, the requirement for mechanical ventilation, the ICU LOS, and the total hospital LOS, each measured in days.

Literature Search Strategy

This meta-analysis was conducted in accordance with the PRISMA 2020 guidelines. A comprehensive literature search was performed to identify studies evaluating the association between baseline vitamin D levels and clinical outcomes in adult burn patients. Searches were conducted in PubMed, Google Scholar, and the Cochrane Library, with additional exploration of grey literature and academic theses using institutional repositories and Google Scholar to capture relevant unpublished data. The search strategy was developed according to the PECO framework, which is suitable for observational studies examining the relationship between an exposure and outcomes. The population comprised adult patients (≥18 years) with burn injuries, including thermal, flame, or electrical burns. The exposure of interest was serum 25-hydroxyvitamin D [25(OH)D] levels measured at the time of admission or before any supplementation, and the comparison was made between patients with sufficient vitamin D levels and those who were insufficient or deficient. The clinical outcomes of interest included infection rates (such as wound infection, bacteremia, and sepsis), ICU admission, ICU LOS, total hospital LOS, and the requirement for mechanical ventilation. The following search terms were applied and adapted for each database: “vitamin D,” “25-hydroxyvitamin D,” “25(OH)D,” “vitamin D deficiency,” “hypovitaminosis D,” “burns,” “burn injury,” “thermal injury,” “burn patients,” “clinical outcome,” “infection,” “sepsis,” “mortality,” “length of stay,” “ICU stay,” “mechanical ventilation,” and “graft loss.” Boolean operators (AND, OR) were used to combine search terms appropriately. No language restrictions were applied to maximize inclusivity; however, all studies that ultimately met inclusion criteria and were available in full text were published in English, and therefore, no eligible non-English studies were included. The final search was conducted on April 12, 2025, yielding 18 results from PubMed, 5,370 from Google Scholar, and four from the Cochrane Library.

Selection of Studies

**Figure 1:**
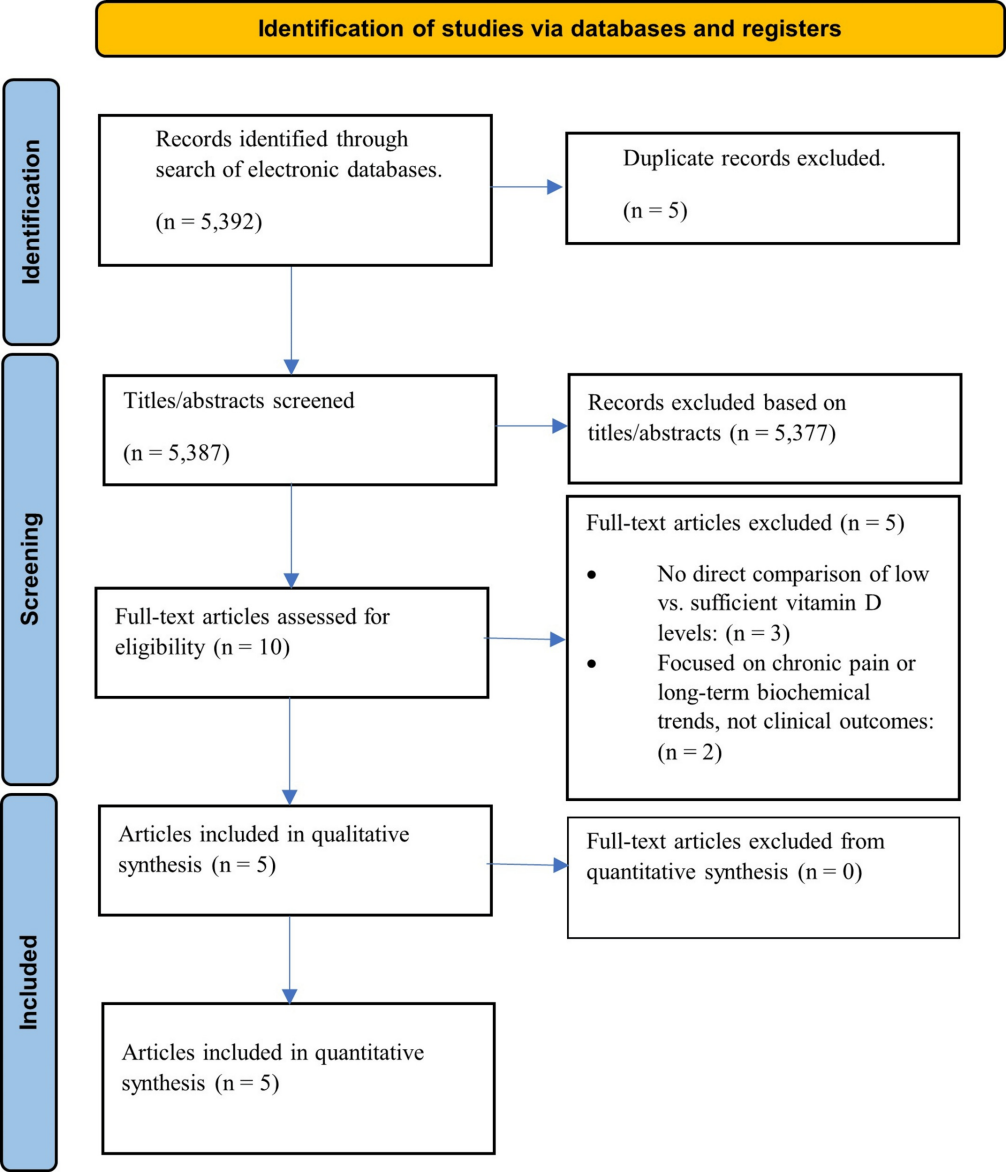
PRISMA flow diagram of study identification, screening, and inclusion for the meta-analysis PRISMA, Preferred Reporting Items for Systematic Reviews and Meta-Analyses

The study selection and data extraction processes were independently conducted by two authors (D.M. and J.P.) (Figure 1). Using the comprehensive search strategy outlined earlier, titles and abstracts of potential literature were identified. Articles deemed suitable during this initial screening underwent full-text review to determine their compliance with our predefined eligibility criteria. Discrepancies in this process were resolved through discussion between the authors. If disagreement persisted, a third author (A.K.) was consulted.

Data Extraction and Management

To ensure systematic and consistent data collection, an electronic data extraction spreadsheet was developed to capture key study details. Two independent authors extracted information from all included studies, encompassing study-related characteristics such as the first author, publication year, country of origin, journal, and study design. Baseline demographic and clinical variables were also recorded, including sample size, vitamin D deficiency cut-off level, timing of blood collection, mean patient age, body mass index, smoking status, history of heart disease, hypertension, diabetes mellitus, malignancy, immunocompromised status, burn severity, burn etiology, and the presence of inhalation injury. All relevant study outcomes were extracted for subsequent quantitative and qualitative synthesis.

Assessment of Risk of Bias

In this meta-analysis, all included studies were observational in nature, employing either retrospective or prospective cohort designs. Accordingly, the Risk of Bias in Non-randomized Studies of Interventions (ROBINS-I) tool was used to evaluate the methodological quality and internal validity of each study. This tool assesses bias across seven key domains: confounding bias (inadequate control of potential confounding variables), selection bias (participant selection and eligibility criteria), classification bias (misclassification of exposure or comparator status), performance bias (deviations from intended exposures or interventions), attrition bias (incomplete outcome data or loss to follow-up), measurement bias (outcome assessment), and reporting bias (selective reporting of results) [18].

Each study was independently assessed by two reviewers, and any disagreements were resolved through consensus. Particular attention was paid to how vitamin D status was defined and measured, whether outcome assessors were blinded to vitamin D status, and whether relevant confounders (e.g., age, burn severity, and comorbidities) were accounted for in the study design or analysis. The risk of bias assessment was applied systematically to ensure a consistent and objective evaluation across all included studies, thereby enhancing the reliability and interpretability of the meta-analysis.

Summary Measures, Outcome Synthesis, and Sensitivity Analysis

We employed Review Manager 5.4.1 (RevMan, Version 5.4.1, Copenhagen, 2020) software to construct a meta-analysis model for comparing outcomes. A random-effects model was used to account for expected heterogeneity across studies. For dichotomous outcomes, we calculated odds ratios (ORs) with corresponding 95% CI, while for continuous outcomes, we used the mean difference (MD) with 95% CI. The ORs represented the odds of an adverse clinical event occurring in patients with low or deficient vitamin D levels compared to those with sufficient vitamin D levels. For continuous variables, the MDs reflected the average difference in duration (e.g., hospital or ICU stay in days) between the same comparison groups.

To evaluate statistical heterogeneity among studies, we calculated and reported the I² statistic and used the Cochran Q test (χ²). Heterogeneity was categorized based on I² values, with low heterogeneity defined as I² ranging from 0% to 25%, moderate heterogeneity from 25% to 75%, and high heterogeneity when I² exceeded 75%. Statistical significance was established using a 95% CI.

To explore potential sources of heterogeneity and assess the robustness of our results, sensitivity analyses were performed. For each outcome parameter, we repeated the meta-analysis using both random-effects and fixed-effect models to evaluate the consistency of results. Additionally, we conducted a leave-one-out sensitivity analysis, iteratively excluding one study at a time to determine the influence of individual studies on the overall pooled estimate and heterogeneity.

Results

The initial search across multiple electronic databases yielded a total of 5,392 articles (Figure 1). After duplicate removal, 5,387 records remained for title and abstract screening. Of these, 5,377 were excluded based on irrelevance to the study objectives or lack of clinical outcome data. The full texts of the remaining 10 articles were retrieved and assessed for eligibility. Following full-text review, five articles were excluded: three did not include a comparison between low and sufficient vitamin D levels, while two focused on unrelated endpoints such as chronic pain or biochemical trends without reporting on acute clinical outcomes relevant to burn care.

Ultimately, five studies were deemed suitable for inclusion in both the qualitative and quantitative synthesis. These studies included adult patients with burn injuries and stratified outcomes based on baseline serum vitamin D status, allowing for comparison between deficient and sufficient groups.

This resulted in a final quantitative synthesis comprising five studies, encompassing a total of 1,814 patients (Figure 1). Among these, 481 patients were classified as having sufficient vitamin D levels, while 1,333 were vitamin D deficient or insufficient based on the definitions provided by the respective studies. Definitions of vitamin D deficiency varied between studies, with most (Zavala et al. [12], Cho et al. [16], and Garner et al. [13]) applying a threshold of <20 ng/mL, while others (Blay et al. [15] and Pirdastan et al. [14]) used <30 ng/mL. These differences may reflect variation in regional guidelines or study populations, as the studies were conducted across diverse geographic and cultural contexts, including the United States, South Korea, and Iran. All included studies reported on at least one of the following outcomes: incidence of wound infection or cellulitis, incidence of bacteremia or septicemia, ICU admission rate, requirement for mechanical ventilation, ICU LOS (days), and total hospital LOS (days), making them suitable for inclusion in meta-analysis.

Detailed information about each study, including authorship, year of publication, country of origin, and study design, is summarized in Table 1. Table 2 presents the baseline demographic and clinical characteristics of the study populations, including sample size, mean age, sex distribution, and where available, TBSA burned and comorbidity data. Outcome-specific data used in the meta-analysis are provided in Table 3, grouped by dichotomous and continuous variables as per the analysis model.

**Table 1:** Study-related data

Author	Year	Country	Journal	Type of study
Blay et al. [15]	2017	USA	Journal of Burn Care & Research	Retrospective observational study
Zavala et al. [12]	2020	USA	Burns	Retrospective observational study
Cho et al. [16]	2020	South Korea	Burns & Trauma	Retrospective observational study
Garner et al. [13]	2022	USA	Burns	Retrospective observational study
Pirdastan et al. [14]	2024	Iran	International Wound Journal	Prospective observational study

**Table 2:** Baseline characteristics of the included population NVD, normal vitamin D; LVD, low vitamin D; BMI, body mass index; HTN, hypertension; DM, diabetes mellitus; NR, not reported; TBSA, total body surface area

Author	No. of Patients (NVD/LVD)	Vitamin D deficiency cut-off (ng/mL) (NVD/LVD)	Baseline Vitamin D (ng/mL) (NVD/LVD)	Collection time	Mean age (%) (NVD/LVD)	BMI (%) (NVD/LVD)	Smoking (%) (NVD/LVD)	Heart disease (%) (NVD/LVD)	HTN (%) (NVD/LVD)	Diabetes (%) (NVD/LVD)	Malignancy (%) (NVD/LVD)	Immunocompromised (%) (NVD/LVD)	Burn severity (%) (NVD/LVD)	Burn etiology (%) (NVD/LVD)	Inhalation Injury (%) (NVD/LVD)
Blay et al., 2017 [15]	318 (65/253)	<30 ng/mL	34.2:16.6 (p≤0.001)	Admission	39.2:43.4 (p=0.086)	25.5:27.3 (p=0.087)	NR	1.5:2.8 (p=>0.999)	1.5:4.3 (p=0.471)	7.7:1.2 (p=0.01)	12.3:6 (p=0.105)	18.5:13.4 (p=0.304)	TBSA burned (median %) 4:5 (p=0.137); partial-thickness injury (%) 89.2:87.6 (p=0.816); full-thickness injury (%) 32.3:42.6 (p=0.137)	Flame (%) 46.2:43.9 (p=0.316); flash (%) 21.5:12.4 (p=0.316); scald (%) 18.5:25.7 (p=0.316); electrical (%) 3.1:2.4 (p=0.316); contact (%) 9.2:10.3 (p=0.316); chemical (%) 0:4 (p=0.316); steam (%) 1.5:1.6 (p=0.316)	NR
Zavala et al., 2020 [12]	107 (44/63)	20 ng/mL	NR	Admission	50:49 (p=0.80)	27.1:27.3 (p=0.38)	NR	4.5:6.3 (p=0.69)	30:27 (p=0.77)	15.9:9.5 (p=0.32)	4.5:9.5 (p=0.34)	0:1.6 (p=0.40)	TBSA burned (median %) 12.1:14.6 (p=0.23)	Flame (%) 70.5:79.4 (p=0.27); scald (%) 25:11.1 (p=0.27); electrical (%) 2.3:1.6 (p=0.27); contact (%) 2.3:4.8 (p=0.27); chemical (%) 0:3.2 (p=0.27)	11.36:11.11 (p=0.97)
Cho et al., 2020 [16]	757 (84/673)	<20 ng//mL	NR	Rehabilitation phase	39.4:39.5	23.5:23.4	6:21.5	NR	NR	NR	NR	NR	TBSA burned (median %) 16.5:21	NR	NR
Garner et al., 2022 [13]	412 (178/234)	<20 ng/mL	NR	Within first 7 days	51.5:49 (p=0.243)	27.1:28.4 (p=0.009)	NR	NR	NR	NR	NR	NR	TBSA burned (median %) 12.2:15 (p=0.002)	Flame (%) 73.2:75.2 (p=0.378); scald/grease (%) 16.2:17.9 (p=0.378); other (%) 10.6:6.8 (p=0.378)	9:15 (p=0.095)
Pirdastan et al., 2024 [14]	220 (110/110)	<30 ng/mL	NR	Admission	44.07:42.30 (p=0.661)	23.73:25.20 (p=0.127)	33.6:30.9 (p=0.665)	0:11 (p=0.58)	33.3:12.5 (p=0.32)	1.1:14.3 (p=0.26)	NR	NR	TBSA burned (median %) 30.62:26.60 (p=0.688)	NR	11.8:16.4 (p=0.688)

**Table 3:** Outcome data Data are presented as NVD:LVD. For dichotomous outcomes (bacteremia/septicemia, ICU admission, and inpatient death), values represent the number of patients. NVD, normal vitamin D; LVD, low vitamin D; UTI, urinary tract infection; LOS, length of stay; ICU, intensive care unit; NR, not reported

Study	Bacteremia/septicemia (NVD:LVD)	UTI (%) (NVD:LVD)	Pneumonia (%) (NVD:LVD)	Cardiovascular complications (%) (NVD:LVD)	Graft loss (%) (NVD:LVD)	Renal failure (%) (NVD:LVD)	Wound infection or cellulitis (%) (NVD:LVD)	Hospital LOS (days) (NVD:LVD)	ICU LOS (days) (NVD:LVD)	ICU admission (NVD:LVD)	Operations (median number) (NVD:LVD)	Ventilator days (NVD:LVD)	Ventilator-free days in the first 28 days (NVD:LVD)	Patients undergoing mechanical ventilation (NVD:LVD)	Inpatient death (NVD:LVD)	Depression (NVD:LVD) (%)	Pain (NVD:LVD) (%)	Itching (NVD:LVD) (%)	Wound healing time (days) (NVD:LVD)
Blay et al., 2017 [15]	3 : 28	1.5:7.1	4.6:11.2	1.5:2.8	1.5:5.1	1.5:1.2	1.5:1.6	2±5.93:3±9.63	2±5.56:8±15.56	19:78	NR	1 (1-11):8 (2-15)	NR	13:61	NR	NR	NR	NR	NR
Zavala et al., 2020 [12]	2 : 10	NR	NR	NR	NR	NR	NR	11.5±9.63:19±19.26	5±12.96:17±20	40:57	1 (0-1):1 (0-2)	NR	28 (27-28):26 (18-28)	NR	2:4	NR	NR	NR	NR
Cho et al., 2020 [16]	NR	NR	NR	NR	NR	NR	NR	62.3±41.4:89.4±54.2	NR	NR	NR	NR	NR	NR	NR	1.2:2.4	15.5:28.2	2.4:6.11	57.4±41.3:84.2±54.3
Garner et al., 2022 [13]	4:36	6.2:12.4	10.7:23.1	NR	1.1:8.1	1.7:7.3	22.5:27.5	12±8.52:18±20	NR	NR	NR	NR	28 (27-28):28 (21-28)	47:98	5:7.5	NR	NR	NR	NR
Pirdastan et al., 2024 [14]	NR	NR	NR	NR	NR	NR	23.6:86.4	11.45±3.32:12.53±3.69	2.4±1.14:3.32±1.43	20:49	4.75±4.83:4.24±3.50	NR	NR	0:7	NR	NR	NR	NR	NR

Assessment of Risk of Bias in Included Studies

**Figure 2:**
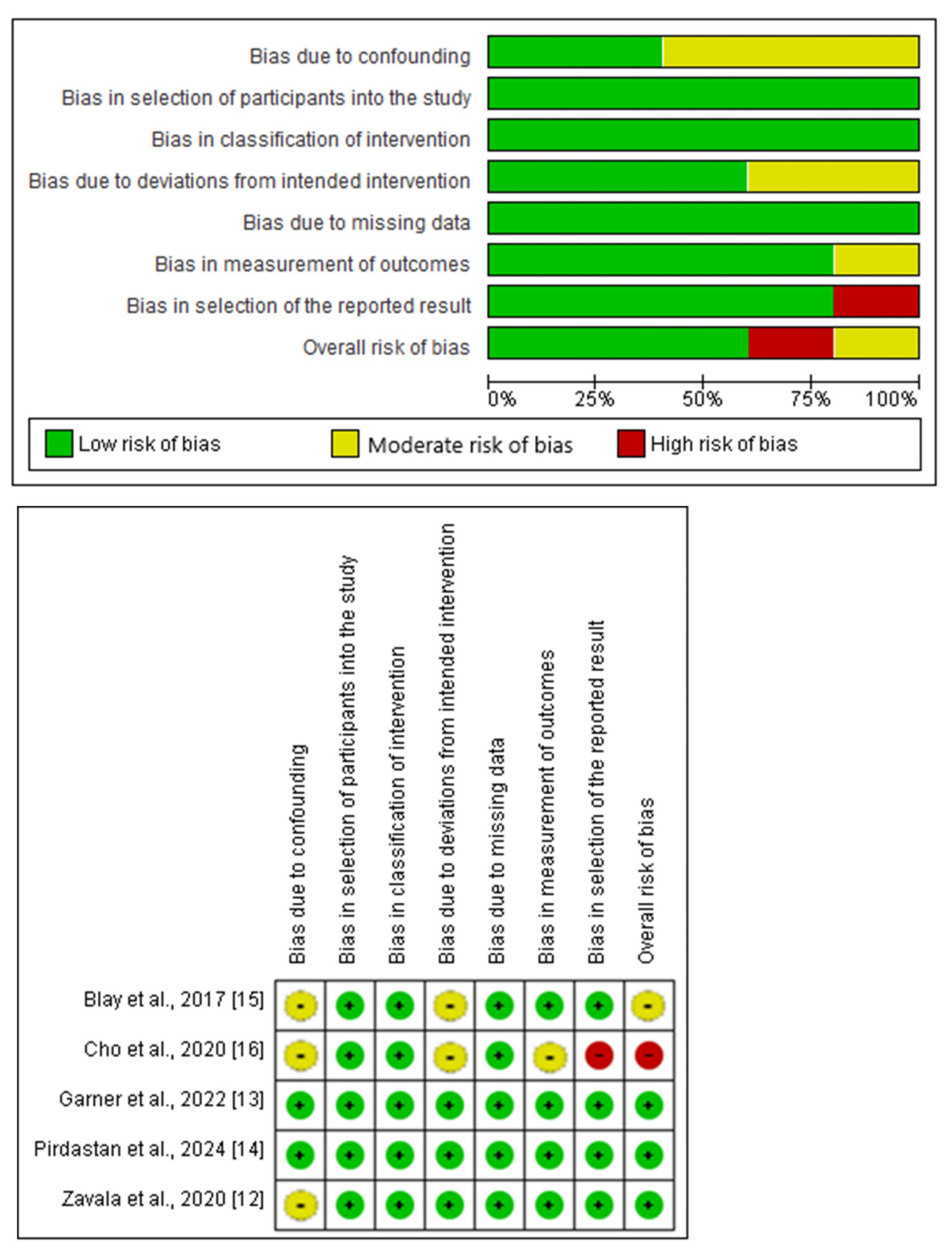
Risk of bias summary and graph showing authors’ judgments for each item, based on the ROBINS-I tool for the included observational studies ROBINS-I, Risk of Bias in Non-randomized Studies of Interventions

The outcomes of risk of bias assessment using the ROBINS-I tool are presented in Figure 2.

Data Synthesis

**Figure 3:**
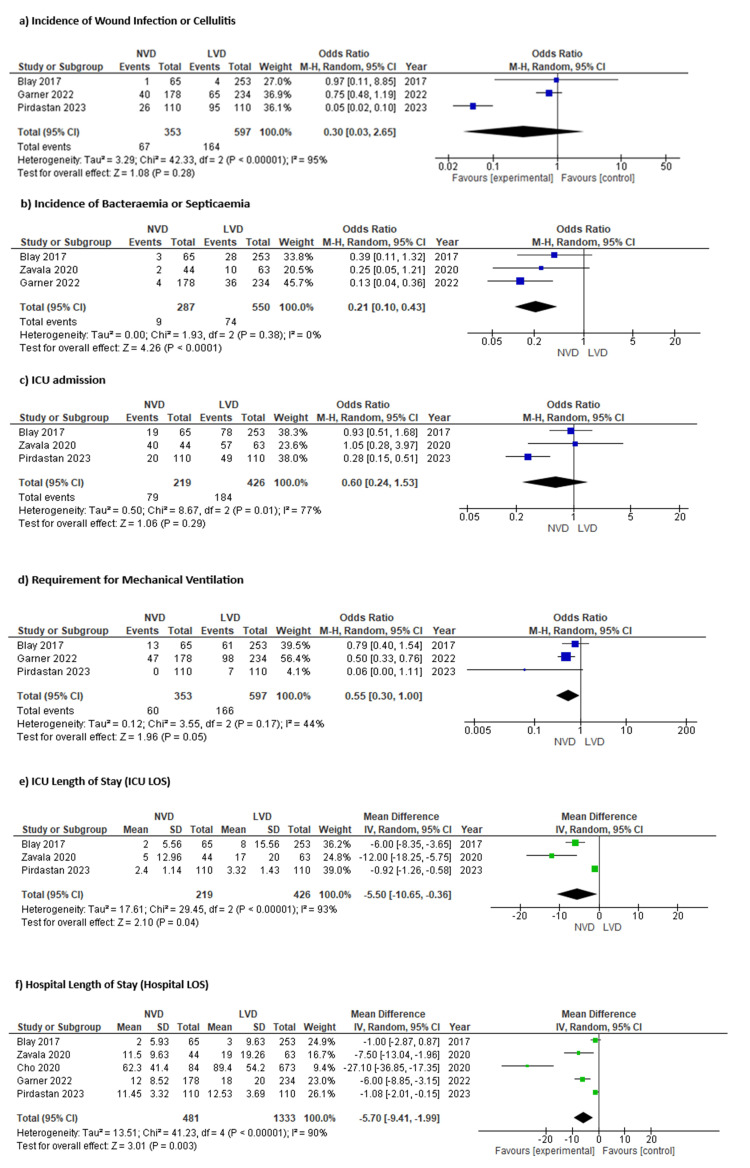
Forest plots of (a) incidence of wound infection or cellulitis, (b) incidence of bacteremia or septicemia, (c) ICU admission, (d) requirement for mechanical ventilation, (e) ICU LOS, and (f) hospital LOS Studies included: Zavala et al., 2020 [12]; Garner et al., 2022 [13]; Pirdastan et al., 2024 [14]; Blay et al., 2017 [15]; Cho et al., 2020 [16]. NVD, normal vitamin D; LVD, low vitamin D; LOS, length of stay; ICU, intensive care unit

Outcomes are summarised in Figure 3.

Incidence of Wound Infection or Cellulitis

The incidence of wound infection or cellulitis was assessed across three studies [13-15], encompassing a total of 950 patients. Among these, 353 patients had normal vitamin D (NVD) levels, and 597 were vitamin D deficient. A total of 67 wound infection or cellulitis events occurred in the NVD group, compared to 164 events in the low vitamin D group.

The meta-analysis demonstrated no statistically significant difference in infection rates between groups, with a pooled OR of 0.30 (95% CI: 0.03 to 2.65, p=0.28), using a random-effects model. However, heterogeneity across the studies was high (I²=95%).

This finding suggests that, while there may be a trend toward a lower risk of wound infection in patients with sufficient vitamin D levels, the variability between studies precludes a definitive conclusion in this outcome.

Incidence of Bacteremia or Septicemia

The incidence of bacteremia or septicemia was assessed across three studies [12,13,15], encompassing a total of 837 patients. Among these, 287 patients were in the NVD group and 550 in the low vitamin D group. A total of nine bacteremia events occurred in the NVD group, compared to 74 in the low vitamin D group.

The meta-analysis demonstrated a statistically significant reduction in the odds of bacteremia among patients with sufficient vitamin D levels, with a pooled OR of 0.21 (95% CI: 0.10 to 0.43, p<0.0001), using a random-effects model. Heterogeneity across studies was low (I²=0%).

This result suggests that vitamin D sufficiency may be associated with a significantly lower risk of systemic infection, such as bacteremia or septicemia, in burn patients.

Intensive Care Unit Admission

The incidence of ICU admission was analyzed across three studies [12,14,15], including a total of 645 patients. Of these, 219 were in the NVD group and 426 in the low vitamin D group. ICU admission was required in 79 patients with sufficient vitamin D levels and 184 patients with vitamin D deficiency.

The pooled analysis demonstrated no statistically significant difference in ICU admission rates between the two groups, with a pooled OR of 0.60 (95% CI: 0.24 to 1.53, p=0.29), using a random-effects model. Heterogeneity across the studies was high (I²=77%).

This suggests that although there may be a trend toward reduced ICU admissions among patients with sufficient vitamin D levels, the inconsistency between studies limits the strength of this finding.

Requirement for Mechanical Ventilation

The need for mechanical ventilation was analyzed across three studies [13-15], involving a total of 950 patients. Of these, 353 were in the NVD group, and 597 were in the low vitamin D group. Mechanical ventilation was required in 60 patients with sufficient vitamin D levels and in 166 patients with low vitamin D levels.

The pooled analysis suggested a non-significant trend toward reduced need for mechanical ventilation in patients with sufficient vitamin D levels, with a pooled OR of 0.55 (95% CI: 0.30 to 1.00, p=0.05), using a random-effects model. Heterogeneity across studies was moderate (I²=44%).

Although the association did not reach conventional statistical significance, the data suggest that vitamin D sufficiency may potentially be associated with reduced need for mechanical ventilation in burn patients, warranting further investigation.

Intensive Care Unit Length of Stay

ICU LOS was assessed across three studies [12,14,15], including a total of 645 patients, with 219 in the NVD group and 426 in the low vitamin D group. All studies reported ICU stay as a continuous variable measured in days.

The pooled analysis showed that ICU stay was significantly shorter in patients with sufficient vitamin D levels compared to those with deficiency. The weighted mean difference (WMD) was -5.50 days (95% CI: -10.65 to -0.36, p=0.04), using a random-effects model. Heterogeneity across studies was high (I²=93%).

These findings suggest that sufficient vitamin D levels may be associated with a shorter duration of ICU stay in patients with burn injuries.

Hospital Length of Stay

Hospital LOS was reported across five studies [12-16], comprising a total of 1,814 patients, with 481 patients in the NVD group and 1,333 in the low vitamin D group. All studies reported hospital stay in days as a continuous outcome.

The pooled analysis demonstrated that patients with sufficient vitamin D levels had a significantly shorter hospital stay compared to those with low vitamin D. The WMD was -5.70 days (95% CI: -9.41 to -1.99, p=0.003), using a random-effects model. Heterogeneity among studies was high (I²=90%).

This result indicates that sufficient vitamin D levels may be associated with a reduction in overall hospitalization duration following burn injury, although the considerable heterogeneity warrants cautious interpretation.

Discussion

Burn injuries remain a critical challenge in acute care, often leading to prolonged hospitalizations, infectious complications, and the need for intensive interventions such as mechanical ventilation and ICU admission [9]. In this context, vitamin D has emerged as a potentially modifiable factor due to its immunomodulatory and wound-healing properties [6,7]. While vitamin D deficiency is prevalent among burn patients [15], its precise role in influencing clinical outcomes remains unclear. However, preclinical animal studies have begun to elucidate plausible mechanisms. In a murine model, Chen et al. [19] demonstrated that administration of 1,25-dihydroxyvitamin D3 significantly reduced early mortality following severe burn injury by alleviating endotoxemia, oxidative stress, and lung inflammation while preserving intestinal barrier integrity. Complementing this, Wu et al. [20] showed that vitamin D deficiency hindered cutaneous wound healing in mice by disrupting the inflammatory response, delaying epithelial-mesenchymal transition (EMT), and impairing extracellular matrix (ECM) remodeling, effects that were partially reversed by vitamin D supplementation. These findings support a potential mechanistic rationale for the observed clinical associations and further emphasize the therapeutic promise of vitamin D in the context of burn injury. This meta-analysis sought to clarify this relationship by synthesizing existing data to determine whether baseline vitamin D status correlates with key clinical endpoints in adult burn patients.

Across the included studies, outcome data varied considerably, highlighting the need for pooled analysis. For wound infection and cellulitis, Blay et al. [15] and Garner et al. [13] reported no statistically significant differences between vitamin D groups, while Pirdastan et al. [14] found a significant increase in the deficient group. In terms of bacteremia and septicemia, all three relevant studies, Blay et al. [15], Zavala et al. [12], and Garner et al. [13], reported a higher incidence in vitamin D-deficient patients, though only Blay et al. [15] and Garner et al. [13] reached statistical significance. For ICU admission, only Pirdastan et al. [14] demonstrated a statistically significant increase among those with low vitamin D levels, while both Blay et al. [15] and Zavala et al. [12] observed non-significant trends in the same direction. With regard to mechanical ventilation, Garner et al. [13] and Pirdastan et al. [14] again reported significant associations with deficiency, whereas Blay et al. [15] did not. ICU LOS was significantly longer in vitamin D-deficient patients in the studies by Blay et al. [15] and Pirdastan et al. [14], but not in Zavala et al. [12]. Finally, for hospital LOS, four studies, Blay et al. [15], Cho et al. [16], Garner et al. [13], and Pirdastan et al. [14], reported significantly prolonged admissions among deficient patients, while Zavala et al. [12] did not. Variability in study design, cohort characteristics, and definitions of vitamin D deficiency contributed to the inconsistencies observed across individual studies in both effect size and statistical significance. These methodological and population-level differences limited the interpretability of findings in isolation and underscored the value of a meta-analytic approach to integrate the evidence. However, such heterogeneity also warrants caution in interpreting even statistically significant associations, as underlying differences between studies may still influence the pooled estimates. To the best of our knowledge, this is the first meta-analysis conducted on this subject, further highlighting the novelty and importance of this review in addressing an emerging clinical question.

This meta-analysis revealed several outcome patterns in relation to vitamin D status among burn patients. Notably, vitamin D sufficiency was significantly associated with lower odds of bacteremia or septicemia. This was the most consistent finding across the included studies, with no observed heterogeneity (I²=0%) and effect sizes in the same direction. This outcome was reported in three retrospective observational studies conducted in the United States (Blay et al. [15], Zavala et al. [12], and Garner et al. [13]), all of which demonstrated higher rates of bloodstream infection in vitamin D-deficient patients. While the definition of deficiency varied slightly (two studies [12,13] used <20 ng/mL and one [15] used <30 ng/mL), baseline characteristics, including age, BMI, and comorbidity distribution, were broadly comparable between groups within each study, reducing the likelihood of confounding. The consistency of this association, despite slight methodological variations, strengthens the plausibility of a link between vitamin D status and systemic infection risk in burn patients. A similar trend was observed for mechanical ventilation, where vitamin D sufficiency appeared protective; however, statistical significance was narrowly missed (p=0.05), and moderate heterogeneity was present. In contrast, outcomes such as ICU admission and wound infection or cellulitis did not demonstrate statistically significant differences between groups, though both showed trends favoring sufficient vitamin D levels and were accompanied by high interstudy variability. Regarding continuous outcomes, vitamin D sufficiency was significantly associated with shorter ICU LOS and hospital stay, despite substantial heterogeneity across studies. These findings suggest that vitamin D deficiency may contribute to prolonged critical care needs and systemic complications in burn patients, though variability between individual study results underscores the importance of pooled analysis to clarify these associations.

To assess the reliability of the pooled results and explore the impact of individual studies, we conducted sensitivity analyses using both model comparison and leave-one-out approaches. Across several outcomes, particularly ICU LOS, ICU admission, and mechanical ventilation, the results were highly sensitive to the inclusion of Pirdastan et al. [14], which consistently shifted statistical significance and contributed disproportionately to heterogeneity. Several methodological and clinical factors likely explain this. First, Pirdastan et al. [14] applied a higher vitamin D deficiency threshold of less than 30 ng/mL, whereas most other studies, such as Zavala et al. [12], Cho et al. [16], and Garner et al. [13], used a lower threshold of less than 20 ng/mL. This broader definition may have included patients with only mild vitamin D deficiency, potentially diluting intergroup differences and reducing the magnitude of observed effects. Moreover, Pirdastan et al. [14] did not report baseline vitamin D levels for either group, limiting insight into the degree of deficiency and undermining comparability between study arms. There were also marked imbalances in comorbidities. The prevalence of heart disease was 0% in the NVD group compared to 12.5% in the low vitamin D (LVD) group, while hypertension was reported in 33.3% of the NVD group versus only 12.5% in the LVD group, an inverse pattern not seen in other included studies. Smoking was reported in 33.6% of the NVD group and 30.9% of the LVD group, which was notably higher than in other studies where smoking data were either lower or not reported. Inhalation injury was also more common in Pirdastan et al. [14], affecting 11.8% of the NVD group and 16.4% of the LVD group. These differences indicate a more clinically complex cohort, with risk factors that could influence ICU outcomes independently of vitamin D status. Burn severity, reflected by TBSA affected, was also substantially higher in Pirdastan et al. [14] than in the other studies. The TBSA was 30.6% in the NVD group and 26.6% in the LVD group, compared to ranges of approximately 4% to 15% in other cohorts. These higher values suggest a more severely burned population in which ICU-related outcomes, such as duration of stay and ventilation requirements, are more likely driven by burn extent and associated complications rather than micronutrient status alone. In this context, any protective effect of adequate vitamin D levels may have been masked by the severity of illness. Additionally, the perfectly equal group sizes in Pirdastan et al. [14] (110 in each arm) increased the study’s statistical weight, further amplifying its influence on pooled estimates. The impact of Pirdastan et al. [14] was most evident in the mechanical ventilation outcome. Its removal from the analysis resulted in a statistically significant pooled effect (p=0.01) and reduced heterogeneity (I²=22%), indicating that its inclusion had diluted the overall association and introduced variability. A similar effect was observed in the ICU admission outcome, which was statistically significant under a fixed-effects model (p=0.003) but lost significance under a random-effects model (p=0.29). Excluding Pirdastan et al. [14] reduced heterogeneity from 77% to 0%, clearly demonstrating its outlier effect. In the wound infection and cellulitis outcome, a stark contrast was seen between models. While the fixed-effect model produced a highly significant result (p<0.00001), this was not upheld under the random-effects model (p=0.28), which showed considerable heterogeneity (I²=95%). This suggests that the significance under the fixed-effect model may be misleading, as it does not account for interstudy variability. The random-effects model, which assumes true heterogeneity across studies, offers a more appropriate and conservative estimate in this context. Taken together, these findings underscore the importance of critically appraising methodological consistency, baseline group comparability, and population characteristics when interpreting meta-analytical results. Despite its statistical contribution, Pirdastan et al. [14] introduced meaningful heterogeneity due to differences in vitamin D classification, lack of baseline data, comorbidity burden, smoking prevalence, inhalation injury, and overall burn severity. These factors must be considered when interpreting the pooled outcomes of this review.

Infectious complications remain a significant concern in burn patients, particularly in the context of immune modulation by vitamin D. For urinary tract infections (UTIs), Blay et al. [15] reported a numerically higher incidence among vitamin D-deficient patients, although this difference was not statistically significant. In contrast, Garner et al. [13] found a significantly higher UTI rate in the vitamin D-deficient group. A similar pattern was observed for pneumonia: Blay et al. [15] noted higher rates in the deficient group without statistical significance, while Garner et al. [13] reported a significantly increased incidence in patients with low vitamin D. These findings suggest a potential role for vitamin D in modulating susceptibility to infectious complications after burn injury, although inconsistencies between studies highlight the need for further investigation.

Beyond infectious complications, several studies explored systemic outcomes such as cardiovascular events, renal failure, and inpatient mortality in the context of vitamin D deficiency following burn injury. Blay et al. [15] assessed cardiovascular complications, including myocardial infarction and arrhythmia, as a composite endpoint but found no statistically significant difference between patients with normal versus deficient vitamin D levels. Similarly, Blay et al. [15] did not observe a significant difference in rates of renal failure across groups, although a numerically higher complication rate was noted among vitamin D-deficient patients. In contrast, Garner et al. [13] reported a significantly increased requirement for renal replacement therapy in the vitamin D-deficient group (7.3% vs. 1.7%, p=0.009), suggesting that vitamin D status may influence the severity of renal dysfunction in more critically ill patients. Regarding inpatient mortality, neither Zavala et al. [12] nor Garner et al. [13] identified a significant difference based on vitamin D status. While these findings indicate that vitamin D deficiency may contribute to the progression of organ dysfunction, particularly renal, they also highlight that the effect on mortality remains inconclusive. This reinforces the need for further focused research to clarify vitamin D’s role in the broader systemic response to burn injury.

Wound-related and surgical outcomes were variably reported across studies, showing mixed findings. Blay et al. [15] observed a higher proportion of graft loss in vitamin D-deficient patients compared to those with sufficient levels, though the difference did not reach statistical significance. In contrast, Garner et al. [13] reported a significantly higher rate of graft loss in the vitamin D-deficient group (8.1% vs. 1.1%, p=0.001), reinforcing the potential role of vitamin D in graft viability. Cho et al. [16] further supported the relevance of vitamin D in tissue repair, showing that wound healing time was significantly prolonged in vitamin D-deficient patients across all burn types, with regression analysis confirming an inverse relationship between vitamin D levels and healing duration. Regarding surgical burden, Zavala et al. [12] found a statistically significant, though marginal, difference in the interquartile range for the number of operations (p=0.047), suggesting a slightly greater operative burden in patients with lower vitamin D levels. However, Pirdastan et al. [14] reported no meaningful difference in operative frequency between vitamin D groups. Collectively, these findings indicate a trend toward worse wound-related and surgical outcomes in vitamin D-deficient burn patients, though inconsistencies across studies highlight the need for further targeted investigation.

Vitamin D status appears to influence the respiratory course of burn patients, particularly regarding the duration and need for mechanical ventilation. While Blay et al. [15] observed a non-significant trend toward longer ventilator days in vitamin D-deficient patients (median 8 vs. 1 day, p=0.146), both Zavala et al. [12] and Garner et al. [13] reported statistically significant differences in ventilator-free days within the first 28 days. Zavala et al. [12] found that patients with sufficient vitamin D levels had a median of 28 ventilator-free days versus 26 in the deficient group (p=0.004), while Garner et al. [13] similarly reported fewer ventilator-free days in the vitamin D-deficient group (IQR: 21.0-28.0 vs. 27.0-28.0, p<0.001). These findings suggest that vitamin D sufficiency may facilitate faster respiratory recovery and reduce the need for prolonged ventilatory support in burn patients. This trend was reflected in our meta-analysis, which showed a borderline significant reduction in the odds of requiring mechanical ventilation in vitamin D-sufficient patients (p=0.05), further reinforcing the potential respiratory benefits of adequate vitamin D levels in this population. Notably, this is consistent with findings from the broader non-burn population, where vitamin D has been shown to enhance pulmonary innate immunity, reduce airway inflammation, and improve lung function, with observational studies reporting associations between higher vitamin D levels and fewer respiratory infections, reduced asthma exacerbations, and improved FEV1 scores [21]. Together, these findings suggest that maintaining adequate vitamin D may have widespread respiratory benefits, including but not limited to patients with burn injuries.

Psychosocial and symptom-related outcomes, including depression, pain, and itching, were explored in Cho et al. [16] among patients undergoing rehabilitation after burn injury. While a higher proportion of patients in the vitamin D-deficient group reported symptoms such as depression (2.4% vs. 1.2%), pain (28.2% vs. 15.5%), and itching (6.1% vs. 2.4%), none of these differences reached statistical significance. These findings suggest that although vitamin D deficiency may trend toward higher symptom burden, the associations were not statistically supported in this study. Therefore, any potential link between vitamin D status and these psychosocial or symptom-related outcomes remains uncertain and warrants further targeted investigation.

Despite the insights gained, several limitations of this meta-analysis must be acknowledged. First, all included studies were observational, limiting causal inference and increasing susceptibility to confounding. Although efforts were made to assess and account for bias, residual confounding, particularly from unmeasured factors such as nutritional status, pre-existing inflammatory conditions, or variations in burn center protocols, remains possible. Second, heterogeneity across studies was considerable for several outcomes. This stemmed in part from methodological differences, including varied thresholds for vitamin D deficiency (e.g., <20 ng/mL vs. <30 ng/mL), inconsistent timing of vitamin D measurements (ranging from admission to rehabilitation), and differing definitions of outcomes such as sepsis and infection. Additionally, not all studies provided complete baseline data (e.g., vitamin D levels, comorbidity distribution, and burn severity), and one study, Pirdastan et al. [14], exerted disproportionate influence on pooled estimates due to outlier population characteristics and statistical weight.

When interpreting these findings, it is important to consider the clinical complexity of burn injuries, multifactorial conditions in which TBSA, inhalation injury, and delay in treatment may independently drive outcomes, regardless of micronutrient status. Furthermore, vitamin D deficiency may reflect underlying vulnerabilities such as frailty or socioeconomic deprivation, acting more as a surrogate marker than a direct driver of morbidity. These nuances underscore the need for cautious interpretation of observed associations.

Future research should focus on well-powered, prospective cohort studies and randomized controlled trials to better delineate the therapeutic role of vitamin D in burn care. Trials such as Rousseau et al. [22], which demonstrated improved musculoskeletal outcomes following supplementation, and Ghadimi et al. [23], which showed significant reductions in systemic inflammation and improved wound healing with vitamin D treatment in a randomized controlled setting, provide preliminary evidence. However, broader evidence is still needed. While all included studies in this meta-analysis met the eligibility criteria, it is important to recognize that several, such as Blay et al. [15], Zavala et al. [12], and Garner et al. [13], involved predominantly small- to moderate-sized burns, many of which would not require inpatient care or fluid resuscitation. In contrast, Cho et al. [16] and Pirdastan et al. [14] included more severe burn populations, making their findings more relevant for shaping future clinical research and practice. If vitamin D status is to be routinely assessed or supplemented in burn management, it is likely to be most impactful in the context of major burns, where physiological derangements may influence both vitamin D levels and clinical outcomes. A 2018 review by Al-Tarrah et al. [24] further highlights the limited number of interventional studies addressing vitamin D supplementation in burn patients, despite biologically plausible roles. The authors identify key barriers to progress, including challenges in interpreting vitamin D status during acute illness, uncertainty around optimal dosing regimens, and variability in assay methods. They emphasize the need for standardized measurement using liquid chromatography-tandem mass spectrometry and advocate for further randomized controlled trials targeting both clinical and mechanistic outcomes. These insights reinforce the rationale for continued exploration of vitamin D as a therapeutic adjunct in burn care. Standardizing deficiency thresholds and sampling protocols, while stratifying analyses by TBSA, age, and comorbidity burden, will be critical. Incorporating mechanistic endpoints, such as immune function and tissue regeneration, may also enhance understanding. Ultimately, if a causal relationship between vitamin D and burn outcomes is confirmed, routine assessment and correction of vitamin D status may be warranted as part of international burn care protocols. Given the high prevalence of deficiency, the low cost of testing and supplementation, and the potential for improved outcomes, this represents an important and actionable direction for future research and clinical guideline development.

## Conclusions

This systematic review and meta-analysis provides the first pooled synthesis of available evidence exploring the relationship between vitamin D status and clinical outcomes in adult burn patients. Vitamin D deficiency was associated with significantly higher odds of bacteremia and longer hospital stays, while other outcomes, such as ICU admission and wound infection, showed variable associations across studies. Heterogeneity and methodological differences between studies, particularly regarding vitamin D cut-off values, measurement timings, outcome definitions, and baseline imbalances, limit the strength of causal inference. Nonetheless, this analysis suggests a potentially important role for vitamin D status in burn-related outcomes, warranting further investigation. Future research, including rigorously designed cohort studies and randomized controlled trials, is needed to validate these associations. Informed by the findings of this review, such studies should standardize vitamin D assessment, stratify patients by burn severity (TBSA and burn depth) and comorbidity burden, and explore mechanistic endpoints. These steps will be essential to determine whether routine assessment and correction of vitamin D status should be formally integrated into international burn care protocols.
